# Risk Factors for Infection, Predictors of Severe Disease, and Antibody Response to COVID-19 in Patients With Inflammatory Rheumatic Diseases in Portugal—A Multicenter, Nationwide Study

**DOI:** 10.3389/fmed.2022.901817

**Published:** 2022-06-13

**Authors:** Ana Rita Cruz-Machado, Sofia C. Barreira, Matilde Bandeira, Marc Veldhoen, Andreia Gomes, Marta Serrano, Catarina Duarte, Maria Rato, Bruno Miguel Fernandes, Salomé Garcia, Filipe Pinheiro, Miguel Bernardes, Nathalie Madeira, Cláudia Miguel, Rita Torres, Ana Bento Silva, Jorge Pestana, Diogo Almeida, Carolina Mazeda, Filipe Cunha Santos, Patrícia Pinto, Marlene Sousa, Hugo Parente, Graça Sequeira, Maria José Santos, João Eurico Fonseca, Vasco C. Romão

**Affiliations:** ^1^Rheumatology Department, Hospital de Santa Maria, Centro Hospitalar Universitário Lisboa Norte, Lisbon Academic Medical Center and European Reference Network on Rare Connective Tissue and Musculoskeletal Diseases Network (ERN-ReCONNET), Lisbon, Portugal; ^2^Instituto de Medicina Molecular João Lobo Antunes, Faculdade de Medicina, Universidade de Lisboa, Lisbon, Portugal; ^3^Rheumatology Department, Centro Hospitalar Universitário de São João EPE, Porto, Portugal; ^4^Rheumatology Department, Instituto Português de Reumatologia, Lisbon, Portugal; ^5^Rheumatology Department, Hospital de Egas Moniz, Centro Hospitalar Lisboa Ocidental, Lisbon, Portugal; ^6^Rheumatology Department, Hospital Garcia de Orta, Almada, Portugal; ^7^Rheumatology Department, Hospital de Braga, Braga, Portugal; ^8^Rheumatology Department, Centro Hospitalar do Baixo Vouga and iBiMED, Institute for Biomedicine, University of Aveiro, Aveiro, Portugal; ^9^Local Health Unit of Guarda, Rheumatology Department, Guarda, Portugal; ^10^Rheumatology Department, Centro Hospitalar de Vila Nova de Gaia/Espinho, Vila Nova de Gaia, Portugal; ^11^Rheumatology Department, Centro Hospitalar e Universitário de Coimbra, Coimbra, Portugal; ^12^Rheumatology Department, Unidade Local de Saúde do Alto Minho, Ponte de Lima, Portugal; ^13^Rheumatology Department, Centro Hospitalar Universitário do Algarve, Faro, Portugal

**Keywords:** SARS-CoV-2, COVID-19, antibody response, seroconversion, inflammatory rheumatic diseases

## Abstract

**Objective:**

To identify risk factors for SARS-CoV-2 infection and for severe/critical COVID-19, and to assess the humoral response after COVID-19 in these patients.

**Methods:**

Nationwide study of adult patients with inflammatory RMDs prospectively followed in the Rheumatic Diseases Portuguese Register—Reuma.pt—during the first 6 months of the pandemic. We compared patients with COVID-19 with those who did not develop the disease and patients with mild/moderate disease with those exhibiting severe/critical COVID-19. IgG antibodies against SARS-CoV-2 were measured ≥3 months after infection and results were compared with matched controls.

**Results:**

162 cases of COVID-19 were registered in a total of 6,363 appointments. Patients treated with TNF inhibitors (TNFi; OR = 0.160, 95% CI 0.099–0.260, *P* < 0.001) and tocilizumab (OR 0.147, 95% CI 0.053–0.408, *P* < 0.001) had reduced odds of infection. Further, TNFi tended to be protective of severe and critical disease. Older age, major comorbidities, and rituximab were associated with an increased risk of infection and worse prognosis. Most patients with inflammatory RMDs (86.2%) developed a robust antibody response. Seroconversion was associated with symptomatic disease (OR 13.46, 95% CI 2.21–81.85, *P* = 0.005) and tended to be blunted by TNFi (OR 0.17, 95% CI 0.03–1.05; *P* = 0.057).

**Conclusions:**

TNFi and tocilizumab reduced the risk of infection by SARS-CoV-2. Treatment with TNFi also tended to reduce rates of severe disease and seroconversion. Older age, general comorbidities and rituximab were associated with increased risk for infection and worse prognosis, in line with previous reports. Most patients with RMDs developed a proper antibody response after COVID-19, particularly if they had symptomatic disease.

## Introduction

Infection by severe acute respiratory syndrome coronavirus 2 (SARS-CoV-2) has quickly become a global concern since the end of 2019 (1). Risk factors for infection and worse prognosis have been extensively documented for the general population, such as older age, cardiovascular and respiratory disease (2, 3). Less evidence is available for patients with inflammatory rheumatic and musculoskeletal diseases (RMDs), whether treated or not with immunosuppressors, although observational data is rapidly accruing (4–8).

Patients with RMDs under conventional synthetic (cs), targeted synthetic (ts) or biological (b) disease-modifying anti-rheumatic drugs (DMARDs) have initially been hypothesized to be at increased risk for development or severe forms of coronavirus disease 19 (COVID-19). However, most observational studies failed to demonstrate an increased vulnerability of patients with RMDs, with a cumulative incidence of COVID-19 similar to the general population and most patients exhibiting mild-to-moderate disease course (9, 10). Indeed, it seems that anti-cytokine therapy does not increase the risk for infection or severe disease (10–12). These drugs have even been hypothesized to be protective of critical disease, by counterbalancing the observed hyperinflammatory state with raised levels of IL-6, IL-1, and TNF (13–15). It remains unclear, though, whether patients with inflammatory RMDs, due to the underlying immune dysregulation and/or to the immunosuppressive treatment, can generate a proper humoral immune response following SARS-CoV-2 infection.

In this study we aimed to: identify risk factors for infection by SARS-CoV-2; assess the clinical outcomes of COVID-19; find predictors for severe and critical disease; and evaluate the development of IgG antibodies against SARS-CoV-2 in patients with inflammatory RMDs.

## Materials and Methods

We performed a multicenter observational nationwide study of adult patients with inflammatory RMDs prospectively followed in the Rheumatic Diseases Portuguese Register (Reuma.pt) in the first 6 months of the pandemic in Portugal—from March 2 (first reported case in the country) to September 30. Reuma.pt is a real-life-based nationwide observational registry that captures a large part of patients with inflammatory rheumatic diseases and the vast majority of patients treated with biological therapies (16). Since March 2020, the Portuguese Society of Rheumatology developed a novel module to capture information on clinical manifestations, treatment, and outcome of rheumatic patients infected with SARS-CoV-2. During this period, all centers ensured their patients' regular follow-up, either through physical appointments or teleconsultations. In this initial phase of the pandemic, the overall awareness of physicians to register any case of COVID-19 in Reuma.pt was high. In addition, all centers were specifically invited to participate in this study.

### Study Population

We included all adult patients with inflammatory RMDs registered at Reuma.pt, with confirmed or suspected infection by SARS-CoV-2 between March 2 and September 30 of 2020. A confirmed case of SARS-CoV-2 infection was defined as a positive RT-PCR on samples obtained from the respiratory tract or positive seroconversion for SARS-CoV-2, regardless of patient's symptoms. A suspected case of COVID-19 was defined as presence of fever plus at least one other respiratory symptom (dyspnoea, persistent cough, odynophagia, anosmia, and/or dysgeusia), or presence of one of the previous symptoms after a contact with a confirmed case, in the absence of a RT-PCR test (17).

In order to evaluate risk factors for SARS-CoV-2 infection, we also included all adult patients with RMDs without confirmed or suspected COVID-19, who were evaluated within the same time frame and had at least one appointment registered in Reuma.pt over the period of concern. Moreover, we used samples from age-, sex-, and sampling date-matched donors (1:2 ratio) of a national serology survey, who had confirmed SARS-CoV-2 infection, but not inflammatory RMDs or immunosuppression, to assess differences in the seroconversion rate from patients with inflammatory RMDs. This control group included patients with a wide range of COVID-19 severity, from asymptomatic disease to severe forms requiring hospitalization.

Inflammatory RMDs were divided into two groups: inflammatory joint diseases (juvenile idiopathic arthritis, rheumatoid arthritis [RA], spondyloarthritis, gout, pseudogout, undifferentiated arthritis, polymyalgia rheumatica, RS3PE, adult onset Still disease) and connective tissue diseases/vasculitis (systemic lupus erythematosus [SLE], systemic sclerosis, Sjögren's syndrome, myositis, undifferentiated connective tissue disease, overlap syndromes, antiphospholipid syndrome and systemic vasculitis).

Medication was analyzed individually or in treatment categories defined as: “None;” “Glucocorticoids,” whether or not on concomitant treatment with DMARDs; “csDMARDs,” including methotrexate, sulfasalazine, hydroxychloroquine, leflunomide, mycophenolate, azathioprine, without b/tsDMARDs; “Tumor necrosis factor (TNF) inhibitors,” “rituximab,” and “other b/tsDMARDs” including tocilizumab, ustekinumab, belimumab and *Janus kinase* inhibitors (JAKi), either with or without concomitant csDMARDs.

This study was approved by the Ethics Committee of the Lisbon Academic Medical Center. All patients signed a specific informed consent.

### Determination of Risk Factors for SARS-CoV-2 Infection and Characterization of COVID-19+ Patients

COVID-19+ patients were compared to those who had registered clinical visits in Reuma.pt in the same time frame, but who did not develop COVID (COVID-19–). We assessed demographics, underlying rheumatic disease, disease activity (last evaluation before the pandemic for COVID-19–; at time of SARS-CoV-2 infection for COVID-19+), comorbidities of interest and medication.

### Determination of Predictors for Severe and Critical COVID-19

Demographic and clinical features of patients with mild/moderate COVID-19 were compared with those with severe/critical disease. Severity of COVID-19 was classified based on World Health Organization definition, as follows: mild disease, symptomatic disease without evidence of pneumonia; moderate disease, clinical and/or radiographic signs of pneumonia existed but room-air SpO2 was ≥90%; severe disease, hypoxemic pneumonia and/or need for hospitalization; critical disease, admission to intensive care unit or death (18).

### Antibody Response to SARS-CoV-2 Infection in Patients With RMDs

Patients were asked to collect a blood sample for antibody testing against SARS-CoV-2 at least 3 months after the resolution of infection. All samples were processed in a single center. Considering that SARS-CoV-2 neutralizing antibodies that inhibit viral replication *in vitro* mainly target the receptor-binding domain (RBD) of the virus spike protein (19–21), we quantified IgG antibodies recognizing the RBD using ELISA (through the assay developed by Krammer et al. a format that received FDA emergency approval in April 2020 and is described in detail elsewhere) (8, 22). Seroconversion was presumed for any titer ≥1:50. Blood samples retrieved were frozen and stored at the Lisbon Academic Medical Center biobank (Biobanco-IMM) until serology processing. A control group, described above, was used to compare the frequency of positive anti-SARS-CoV2 IgG and respective titration. Reuma.pt and Biobanco-IMM are both approved by the Ethics Committee of the Lisbon Academic Medical Center and by the Portuguese Data Protection Authority.

### Statistical Analysis

Categorical variables were reported as percentages, whereas continuous variables were expressed as median (IQR). Continuous variables were compared by the use of the Student's two-tailed *t*-test or the non-parametric Mann-Whitney *U*-test as appropriate. Categorical variables were compared using Chi-square or Fisher's exact test. We estimated the odds ratio and 95% confidence intervals (CI) for independent associations between demographic, disease-related and treatment-related variables and the mentioned outcomes, through multivariate logistic regression analysis. Age, sex, and variables associated with the outcome in univariate analysis with a *P*-Value < 0.1 were included in the models with a stepwise backward selection methodology. *P*-Values < 0.05 were considered significant. Statistical analyses were conducted using SPSS IBM v.24, Chicago, IL, United States.

## Results

### Characterization of COVID-19+ Patients

Overall, 157 confirmed and five suspected cases of COVID-19 were recorded in Reuma.pt, out of 6,363 patients with registered visits in the period of interest [median age 54 (IQR 19) years; 74.7% female]. The majority of patients had inflammatory joint diseases (*N* = 111; 68.5%) and 18.5% (30/162) had moderate or high disease activity at the time of infection (Table 1 and Supplementary Table S1). Almost three quarters of the patients (*N* = 120; 74.1%) were under DMARDs (csDMARDs or b/tsDMARDs monotherapy or combination) and 70 (43.2%) were on systemic glucocorticoids. Most patients became infected after direct contact with a positive patient (*N* = 111, 68.5%; Supplementary Table S2) and the most common symptoms (cough, fever, malaise) and laboratory abnormalities (lymphopenia, elevated C-reactive protein) were in line with what was expected (Supplementary Table S2). After confirmed or suspected infection, a minority of patients suspended csDMARDs by their own choice or according to their physician advice (34/97,100, 35.1%), whereas more than half suspended bDMARDs or tsDMARDs (23/40, 57.5%). Most patients under glucocorticoids remained on their usual dosage (57/70, 81.4%). Patients with asymptomatic SARS-CoV2 infection were not different from symptomatic patients regarding age, sex, comorbidities, rheumatic disease or treatment.

**Table 1:** Patient characteristics according to COVID-19 status.

	**COVID-19+ (*N* = 162)**	**COVID-19– (*N* = 6,201)**	***P*** **-value**
Female, *N* (%)	121 (74.7)	4,122 (66.5)	**0.028**
Age (years), median (IQR)	54 (19)	56 (22)	0.707
Caucasian, *N* (%)	136 (83.9)	4,157 (67.0)	**0.001**
Smoking (ever), *N* (%)	31 (19.1)	1,270 (20.5)	**0.025**
**Rheumatic disease by subgroup**^***a***^, ***N*** **(%)**			
Inflammatory joint diseases	111 (68.5)	5,114 (82.5)	**<0.001**
CTD and vasculitis	51 (31.5)	1,087 (17.5)	
**Rheumatic disease by diagnosis**, ***N*** **(%)**			
**Inflammatory joint diseases**			
Rheumatoid arthritis	48 (29.6)	2,430 (39.2)	**<0.001**
Spondyloarthritis	32 (19.8)	1,526 (24.6)	
Psoriatic arthritis	20 (12.3)	942 (15.2)	
Other^*b*^	11 (6.8)	216 (3.5)	
**CTD and vasculitis**			
Systemic lupus erythematosus	12 (7.4)	477 (7.7)	
Vasculitis	8 (4.9)	139 (2.2)	
UCTD	8 (4.9)	46 (0.7)	
Systemic sclerosis	7 (4.3)	213 (3.4)	
Sjögren's syndrome	5 (3.1)	133 (2.1)	
Other^*c*^	11 (6.8)	79 (1.3)	
Disease duration (years), median (IQR)	7.5 (11.1)	10.6 (12.5)	**<0.001**
**Disease activity**^***d***^, ***N*** **(%)**			
Remission	59 (36.4)	1,807 (29.1)	**<0.001**
Low	65 (40.1)	1,061 (17.1)	
Moderate	20 (12.3)	1,200 (19.4)	
High	10 (6.2)	336 (5.4)	
**Comorbidities**, ***N*** **(%)**^***e***^			
≥1 comorbidity^*f*^	65 (40.1)	1,548 (25.0)	**<0.001**
≥2 comorbidities	27 (16.7)	422 (6.8)	**<0.001**
**Ongoing immunosuppressive treatment by class**, ***N*** **(%)**			**<0.001**
None	33 (20.4)	69 (1.1)	**<0.001**
Glucocorticoids	70 (43.2)	2,128 (34.3)	0.371
csDMARDs	80 (49.4)	1,612 (25.9)	**<0.001**
TNF inhibitors	24 (14.8)	2,598 (41.9)	**<0.001**
Rituximab	7 (4.3)	261 (4.2)	1.000
Other b/tsDMARDs	9 (5.6)	716 (11.5)	**0.002**

*^a^For the multivariate analysis, inflammatory joint diseases were set as the reference category; ^b^Other diagnoses: Crystal-induced arthropathies (n = 4), Polymyalgia rheumatica (n = 3), Juvenile idiopathic arthritis (n = 2), Adult-onset Still's disease (n = 1), RS3PE (n = 1); ^c^Other diagnoses: antiphospholipid syndrome (n = 3), Mixed connective tissue disease (n = 3), Sarcoidosis (n = 2); Myositis (n = 2), Overlap syndromes (n = 1); ^d^Disease activity in the last evaluation before the pandemic for COVID-19– patients and at the time of the SARS-CoV-2 infection for the COVID-19+ patients; ^e^Specification of comorbidities and individual drug treatments can be found in Supplementary Table S1. ^f^At least 1 comorbidity among arterial hypertension, obesity, diabetes, cardiovascular disease, asthma, chronic obstructive pulmonary disease, chronic kidney disease. b, biological; cs, conventional synthetic; CTD, connective tissue disease; DMARDs, disease-modifying anti-rheumatic drugs; JAKi, Janus Kinase inhibitors; NS, statistically non-significant; NSAIDs, non-steroidal anti-inflammatory drugs; TNFi, tumor necrosis factor inhibitors; ts, targeted synthetic; UCTD, undifferentiated connective tissue disease. Bold values means statistical significance p value < 0.05*.

### Risk Factors for SARS-CoV-2 Infection

In order to assess risk factors for infection by SARS-CoV-2, we compared the demographic and clinical data of these 162 COVID-19+ patients with 6,201 controls (COVID-19–) (Table 1 and Supplementary Table S1). On univariate analysis, COVID-19+ patients were more frequently female, Caucasian, never smokers and had a higher prevalence of chronic kidney disease. The prevalence of COVID-19 in patients with connective tissue diseases or vasculitis (4.5%) was more than twice that of those with inflammatory joint diseases (2.1%, *P* < 0.001). Further, COVID-19+ patients had a shorter rheumatic disease duration and lower disease activity. Regarding treatment, patients under csDMARDs (*n* = 80/1,692, 4.7%) or b/tsDMARDs (*n* = 40/3,615, 1.1%) were significantly less likely to become infected than those not receiving any immunosuppressive treatment (*n* = 33/102, 32.4%; OR 0.104, 95% CI 0.065–0.166, *P* < 0.001, and OR 0.023, 95% CI 0.014–0.039, *P* < 0.001, respectively), albeit the numbers were small in the latter group. Drug subanalysis (Supplementary Table S1) showed that TNF inhibitors (TNFi) were more frequently used in COVID-19– patients (41.9 vs.14.8% in COVID-19+, *P* < 0.001). Tocilizumab also showed a similar trend (6.0 vs. 2.8% in COVID-19+, *P* = 0.057). On multivariate analysis, moderate/high disease activity and use of TNFi or tocilizumab were independently associated with a lower likelihood of developing COVID-19 (Table 2). On the other hand, being Caucasian or having two or more comorbidities were independent risk factors for SARS-CoV-2 infection.

**Table 2:** Multivariate binary logistic regression model for prediction of COVID-19 infection.

	**OR**	**95% confidence interval**	***P*-value**
Female	1.240	0.821–1.874	0.307
Caucasian	2.548	1.419–4.575	**0.002**
≥2 comorbidities	2.273	1.416–3.649	**0.001**
**Disease activity**			
Remission/low disease activity	Ref.	–	–
Moderate/high disease activity	0.409	0.261–0.640	**<0.001**
**RMD subgroup**			
Inflammatory RMD	Ref.	–	–
CTD and vasculitis	1.051	0.663–1.667	0.833
Methotrexate	0.781	0.532–1.145	0.205
TNF inhibitors	0.160	0.099–0.260	**<0.001**
Tocilizumab	0.147	0.053–0.408	**<0.001**

*AUC = 0.714 95% CI 0.670–0.757. Bold values means statistical significance p value < 0.05*.

### Predictors of Severe/Critical COVID-19

Thirty-seven (22.8%) COVID-19+ patients developed severe/critical disease, requiring hospitalization, 28 (17.3%) needed oxygen supply, 11 (6.8%) non-invasive ventilation and 3 (1.9%) mechanical ventilation (Table 3). A total of 8 (4.9%) patients died. In addition to supportive treatment, hospitalized patients were most commonly treated with hydroxychloroquine (25, 15.4%), glucocorticoids (15, 9.3%), azithromycin (13, 8.0%) and non-specific antivirals (6, 3.7%). Thirty-one patients (19.1%) developed at least one complication, most commonly bacterial superinfection and severe acute respiratory illness (Table 3). Two patients (1 with SLE, 1 with RA) were diagnosed with macrophage activation syndrome and 1 patient with primary Sjögren's syndrome had a non-fatal thromboembolic event.

**Table 3:** Characterization of COVID-19 in patients with RMDs.

	**Overall**
	***N* = 162**
**Type of COVID-19 diagnosis**, ***N*** **(%)**	
PCR confirmed	153 (94.4)
Positive serology	4 (2.5)
Suspected^*a*^	5 (3.1)
**COVID-19 severity**, ***N*** **(%)**	
Asymptomatic	17 (10.5)
Mild	27 (16.7)
Moderate	81 (50.0)
Severe	24 (14.8)
Critical	13 (8.0)
**COVID-19 hospitalization care**, ***N*** **(%)**	
Hospitalization	37 (22.8)
Supplemental oxygen	28 (17.3)
Non-invasive ventilation	11 (6.8)
Invasive ventilation	3 (1.9)
**COVID-19 treatment**, ***N*** **(%)**	
Glucocorticoids	15 (9.3)
Hydroxychloroquine	25 (15.4)
Azithromycin	13 (8.0)
Other antibiotic	5 (3.1)
Lopinavir/ritonavir	4 (2.5)
Remdesivir	2 (1.2)
Tocilizumab	1 (0.6)
Intravenous immunoglobulin	1 (0.6)
**Death from COVID-19**	8 (4.9)
**Other complications**^***b***^, ***N*** **(%)**	
Acute respiratory distress syndrome	11 (6.8)
Heart failure	1 (0.6)
Bacterial infection	15 (9.3)
Macrophage activation syndrome	2 (1.2)
Thromboembolic event	1 (0.6)
Acute kidney failure	6 (3.7)

*^a^Defined in the Methods Section, RT-PCR not performed*.

*^b^Each patient could have more than one complication*.

Compared to patients with mild/moderate COVID-19, those with severe/critical course were older and had a higher prevalence of arterial hypertension, diabetes, cardiovascular disease, and chronic kidney disease (Table 4). Regarding therapy, the proportion of patients under rituximab was higher in severe/critical COVID-19 patients (10.8 vs. 2.4%, *P* = 0.043), while treatment with TNFi was associated with less probability of severe/critical COVID-19 (2.7 vs. 18.4%, *P* = 0.044). Deaths, considered individually, were also more frequent in patients who had arterial hypertension, diabetes, cardiovascular disease, chronic kidney disease and patients treated with rituximab. No differences were found concerning sex, disease activity status before COVID-19, or median time to symptom resolution/negative RT-PCR. On multivariate analysis, age and treatment with rituximab were the only independent factors strongly associated with severe/critical COVID-19 (Table 4).

**Table 4:** Comparison of COVID-19+ patients with severe/critical course and mild/moderate disease.

	**Severe/critical COVID-19** **(*n* = 37)**	**Mild/moderate COVID-19** **(*n* = 125)**	**Univariate analysis OR** **(95%CI); *P*-value**	Multivariate analysis* **OR (95%CI); *P*-value**
Female, *N* (%)	25 (67.6)	96 (76.8)	0.63 (0.28–1.41); 0.259	
Age (years), median (IQR)	68 (24)	52 (18)	**1.09 (1.06–1.13); <0.001**	**1.09 (1.05–1.14); <0.001**
Caucasian, *N* (%)	32 (86.5)	104 (83.2)	0.20 (0.03–1.59); 0.129	
Smoking (ever), *N* (%)	8 (21.6)	23 (18.4)	1.27 (0.50–3.21); 0.613	
Disease duration (years), median (IQR)	10.4 (13.2)	7.1 (10.2)	**1.05 (1.01–1.09); 0.023**	
**Disease activity before COVID-19**, ***N*** **(%)**
Remission	16 (43.2)	43 (34.4)	Ref.	
Low	9 (24.3)	56 (44.8)	0.43 (0.17–1.07); 0.070	
Moderate	5 (13.5)	15 (12.0)	0.90 (0.28–2.87); 0.853	
High	4 (10.8)	6 (4.8)	1.79 (0.45–7.19); 0.411	
**Comorbidities**, ***N*** **(%)**				
No comorbidities	15 (40.5)	82 (65.6)	Ref.	Ref.
≥1 comorbidity^*a*^	22 (59.5)	43 (34.4)	**2.80 (1.32–5.94); 0.007**	1.15 (0.47–2.82); 0.759
**Comorbidities (detailed)**, ***N*** **(%)**				
Obesity	7 (18.9)	23 (18.4)	1.04 (0.41–2.65); 0.943	
Arterial hypertension	15 (40.5)	27 (21.6)	**2.67 (1.21–5.90); 0.015**	
Diabetes	6 (16.2)	3 (2.4)	**8.28 (1.95–35.07); 0.004**	
Cardiovascular disease	7 (18.9)	4 (3.2)	**7.44 (2.04–27.17); 0.002**	
Chronic kidney disease	4 (10.8)	2 (1.6)	**7.46 (1.31–42.49); 0.024**	
Cerebrovascular disease	1 (2.7)	4 (3.2)	0.84 (0.09–7.76); 0.878	
Asthma	2 (5.4)	0 (0.0)	NA; 0.051^*b*^	
Chronic obstructive pulmonary disease	1 (2.7)	4 (3.2)	0.84 (0.09–7.76); 0.878	
Interstitial lung disease	1 (2.7)	1 (0.8)	3.44 (0.21–56.45); 0.386	
Hyperuricemia	1 (2.7)	2 (0.8)	3.44 (0.21–56.45); 0.386	
Malignancy	3 (8.1)	4 (3.2)	2.67 (0.57–12.51); 0.213	
**Immunosuppressive treatment at COVID-19 diagnosis**, ***N*** **(%)**				
None	10 (27.0)	23 (18.4)	1.64 (0.70–3.86); 0.255	
Glucocorticoids	17 (45.9)	53 (42.4)	1.16 (0.55–2.41); 0.702	
csDMARDs	17 (45.9)	63 (50.4)	0.84 (0.40–1.75); 0.634	
TNFi	1 (2.7)	23 (18.4)	**0.123 (0.02–0.95); 0.044**	0.29 (0.04–2.44); 0.291
Rituximab	4 (10.8)	3 (2.4)	**4.93 (1.05–23.12); 0.043**	**9.20 (1.53–55.45); 0.015**
Other b/ts DMARDs^*c*^	0 (0.0)	9 (7.2)	NA; 0.120^*b*^	
**COVID-19 duration (days)**, **median (IQR)**				
Time to symptom resolution	27 (22)	20 (19)	1.01 (0.99–1.03); 0.348	
Time to negative RT-PCR	27.7 (25)	32 (24)	1.00 (0.97–1.03); 0.997	

*^a^At least 1 comorbidity among arterial hypertension, obesity, diabetes, cardiovascular disease, asthma, chronic obstructive pulmonary disease, chronic kidney disease. ^b^Logistic regression not applicable (NA); P-Value state for fisher's test. ^c^Includes tocilizumab (n = 5), ustekinumab (n = 1), belimumab (n = 1), Janus kinase inhibitors (n = 2). CTD, connective tissue diseases; TNFi, tumor necrosis factor inhibitors*.

*Binary logistic regression model for prediction of severe/critical disease. Bold values means statistical significance p value < 0.05.

### Antibody Response After SARS-CoV-2 Infection

**Figure 1:**
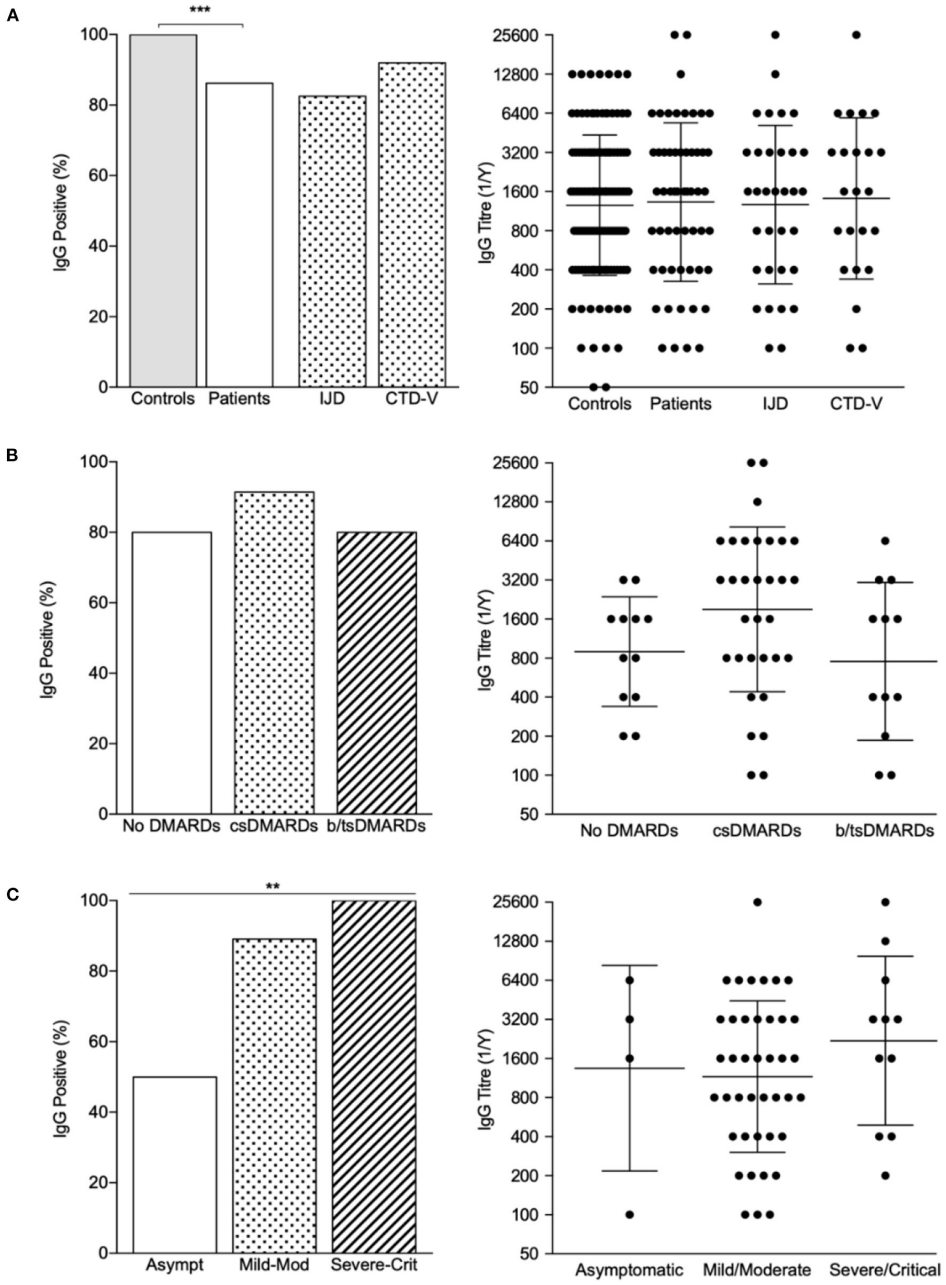
IgG seroprevalence rates and titer distribution across patients and controls. **(A)** Proportion of IgG positive patients and IgG titers in controls and patients, with individualization per rheumatic disease type. ***P < 0.001. **(B)** Proportion of IgG positive patients and IgG titers across treatment classes; bars represent GMT ± GSD. **(C)** Proportion of IgG positive patients and IgG titers according to COVID-19 severity; bars represent GMT ± GSD. **P < 0.01. b/ts, biological/targeted synthetic; cs, conventional synthetic; CTD-v, connective tissue diseases and vasculitis; DMARDs, disease modifying antirheumatic drugs; IJD, inflammatory joint diseases.

Out of the 162 included patients, 65 (40%) performed antibody testing (Figure 1 and Supplementary Table S3). Blood samples were collected between days 89 and 331 (median time 237, IQR 149 days) after symptom onset or positive PCR test (if asymptomatic). Fifty-six (86%) patients had positive IgG antibody titers, with a geometric mean titer of 1/1,329 [geometric SD factor (GSD) 4.063; range 1/100–1/25,600]. In comparison with the age-, sex-, and sample-timing-matched control population (*n* = 130), patients with RMDs (*n* = 65) had a lower seroconversion rate (100.0 vs. 86.2%, *P* < 0.001; Figure 1A and Supplementary Table S4). Nonetheless, antibody titers in subjects with detectable levels did not differ between groups (Figure 1A and Supplementary Table S4).

Regarding RMDs patients who seroconverted, 44 (78.6%) were on cs- or b/tsDMARD therapy (Table 5). All but one (a patient with SLE under hydroxychloroquine who developed fever, cough, myalgias, and anosmia following a high-risk contact, but who tested negative on RT-PCR) were COVID-19 confirmed cases. Regarding the nine patients who did not seroconvert, six were RT-PCR-positive confirmed cases, one tested negative on RT-PCR and two did not perform this test. All of the three latter suspected cases had a high clinical suspicion based on symptoms and exposure history. IgG- patients had mostly mild (*n* = 1 SLE under methotrexate and hydroxychloroquine) or asymptomatic disease (*n* = 4, 3 of whom on hydroxychloroquine, methotrexate or etanercept). Still, 4 patients with moderate disease, presenting clinical/radiological signs of pneumonia (SpO2 ≥90%), also had undetectable antibodies.

**Table 5:** Comparison of COVID-19+ patients based on antibody response.

	**IgG+** **(*n* = 56)**	**IgG–** **(*n* = 9)**	**Univariate analysis** **(*P*-value)**	**Multivariate analysis OR** **(95% CI); *P*-value^***a***^**
Female, *N* (%)	42 (75.0)	6 (66.7)	0.418	
Age (years), median (IQR)	56 (16)	52 (24)	0.649	
**Rheumatic disease group**, ***N*** **(%)**				
Inflammatory joint diseases^*b*^	33 (58.9)	7 (77.8)	0.463	
CTD/Vasculitis	23 (41.1)	2 (22.2)		
**Disease activity before infection**, ***N*** **(%)**				
Remission/low	45 (80.4)	8 (88.9)	0.673	
Moderate/high	9 (16.1)	1 (11.1)		
**Immunosuppressive treatment**, ***N*** **(%)**				
None	12 (21.4)	3 (33.3)	0.420	0.17 (0.03–1.05); 0.057
Glucocorticoids	20 (51.8)	0 (0.0)	**0.003**	
csDMARDs^*c*^	32 (57.1)	3 (33.3)	0.282	
TNFi^*d*^	6 (10.7)	3 (33.3)	0.068	
Rituximab^*e*^	2 (3.6)	0 (0.0)	1.000	
Other bDMARDs^*f*^	4 (10.7)	0 (0.0)	1.000	
**Comorbidities**				
Hypertension	18 (32.1)	1 (11.1)	0.264	
Diabetes mellitus	2 (3.6)	1 (11.1)	0.365	
Cardiovascular disease^*g*^	3 (5.4)	1 (11.1)	0.458	
Obesity	15 (26.8)	2 (22.2)	1.000	
**COVID-19 severity (** ***N*** **, %)**				
Asymptomatic^*h*^	4 (7.1)	4 (44.4)	**0.028**	Ref.
Mild	9 (16.1)	1 (11.1)		13.46 (2.21–81.85); **0.005**
Moderate	32 (57.1)	4 (44.4)		
Severe	10 (17.9)	0 (0.0)		
Critical	1 (1.8)	0 (0.0)		
**Sample timing**^***i***^ **(days) Median (IQR)**	236 (126)	239 (105)	0.875	

*^a^Binary logistic regression model for prediction of IgG development; ^b^Reference for statistical analysis; ^c^In IgG+: azathioprine (n = 4), hydroxychloroquine (n = 12), leflunomide (n = 4), mycophenolate (n = 1), methotrexate (n = 24), sulfasalazine (n = 3), In IgG-: hydroxychloroquine (n = 1), methotrexate (n = 3); ^d^The three patients under TNFi who did not develop IgG antibodies were on monotherapy; ^e^Last dose on day 135 before symptom onset and day 224 before antibody testing for 1 patient with SLE and not specified for the other patient with RA; ^f^Belimumab (n = 1), tocilizumab (n = 2), ustecinumab (n = 1); ^g^Includes personal history of heart failure and/or ischemic cardiopathy. ^h^Reference for statistical analysis. ^i^Relative to symptom onset or first positive RT-PCR test if asymptomatic. CTD, connective tissue diseases; TNF, tumor necrosis factor inhibitors. Bold values means statistical significance p value < 0.05*.

Age, sex, and disease activity status at the time of the infection did not influence seroconversion (Table 5). Although DMARD therapy as a whole did not influence seropositivity rate, the proportion of patients on TNFi was numerically higher in patients who did not develop IgG antibodies (33.3 vs. 10.7%) (Figure 1B and Table 5). Of note, all patients treated with corticosteroids (*n* = 20) and rituximab (*n* = 2) developed antibodies. There was no correlation between sample timing and anti-RBD IgG titers (Spearman *r* = −0.042, *P* = 0.755). On multivariate analysis, symptomatic COVID-19 was strongly associated with the development of serological response (Figure 1C and Table 5). In turn, TNFi treatment tended to negatively impact seroconversion (Table 5).

## Discussion

During the first months of the pandemic, the European contagion wave hit Portugal with a slight delay, allowing the creation of a dedicated, structured and prospective module in Reuma.pt aiming to assess COVID-19 consequences in patients with RMDs. We took advantage of this effort to provide a broad characterization of the SARS-CoV-2 infection in this vulnerable patient population.

We found that Caucasian ancestry and having ≥2 comorbidities were risk factors for infection, while moderate/high RMD disease activity status and treatment with TNFi and tocilizumab were protective. The association of Caucasian ancestry with COVID-19 was unexpected, considering that most studies assessing ancestral and ethnic disparities pointed to a higher prevalence of the disease in Hispanics and Blacks, possibly related to a lower socioeconomic status and a higher comorbidity burden (23, 24). It is possible that other social, cultural, and genetic factors of the Portuguese population contribute to this discrepancy. Regarding disease activity, we may argue that patients with RMDs with more active disease were more aware of the disease itself, as well as of the associated immune dysregulation and drug-related immunosuppression. Thus, they might have complied with measures of social distancing and hygiene in a more scrupulous way. However, it is not possible to exclude a true protective role of some DMARDs, namely TNFi and tocilizumab. Indeed, this is an exciting finding, that is reported in a large population of RMD patients treated with TNFi (*n* = 2,622). These targeted therapies are used to treat both inflammatory diseases with more aggressive phenotypes, and severely ill patients with COVID-19, considering the major contribution of TNF and IL-6 to the cytokine-storm syndrome observed in critical patients (13, 14, 25). Similarly to our findings, a smaller single center retrospective study reported that COVID-19+ patients were less likely treated with IL6-inhibitors (26). Likewise, Simon et al. (27) reported a significantly lower SARS-CoV-2 seroprevalence in 534 patients with immune-mediated inflammatory diseases (IMIDs; 295 of whom had RMDs) receiving targeted anti-cytokine therapies compared to healthy individuals (RR 0.32; 95% CI 0.11–0.99). This was not observed in patients with IMIDs not receiving immunosuppressors, who exhibited similar seroprevalence to controls (27). We have expanded these results in a considerably larger population (*n* = 3,615 RMD patients treated with b/tsDMARDs). Although these results do not prove that TNFi or anti-IL6 bDMARDs prevent SARS-CoV-2 infection, attending to limitations in the study design and difficulty to control confounders factors (e.g., different protective behaviors and patterns of corticosteroids use among patients treated with bDMARDs), these are reassuring data and may guide clinicians regarding the decision-making process on which biologic to initiate during the pandemic.

As of 30 September 2020, the overall rate of COVID-19-associated hospitalization and mortality in Portugal were, respectively, 8.4 and 2.6% (28). In comparison, the population with RMDs exhibited a strikingly higher burden of the disease-−22.8% (37/162) were hospitalized and 4.9% died. Previous studies also found similar results, such as those reported by the Italian cohort, which was significantly affected by the COVID-19 pandemic during the first wave −9% of patients with RMDs died, compared to 4% of the general population [OR 3.10 (95% CI 2.29–4.12)] (29), between March and November 2020. Although a report bias must be taken into account, this greater risk for worse outcomes may also be explained by a compromised health status and iatrogenesis posed by some baseline therapies. Rituximab is particularly relevant in this regard, as it was the only DMARD independently associated with the risk of severe/critical COVID-19 in our analysis, in line with previous reports (5, 30, 31), including a recent systemic review (SLR) and meta-analysis (32). In the opposite direction, and in addition to the discussed beneficial effect in reducing COVID-19 risk, TNFi were negatively associated with severe/critical disease, suggesting a potential protective role against the combined outcome of hospitalization and/or death. This observation, though, was not confirmed in the multivariate model. Nevertheless, our results are in accordance with those reported in the Global Rheumatology Alliance (GRA) landmark paper including 600 COVID-19+ patients from 40 countries, 46% of whom required hospitalization (4). Therein, older age, comorbidities and prednisone-equivalent ≥10 mg/day were predictors for hospitalization, whereas b/tsDMARD monotherapy showed a protective role, an effect mainly driven by TNFi. Finally, contrary to others authors (5, 33, 34), we could not confirm a deleterious prognostic role of glucocorticoids and higher disease activity. This may be attributed to other relevant factors such as disease/treatment heterogeneity between studies, variegated COVID-19 treatment protocols, and potential genetic/environmental differences across populations.

Concerning antibody response, to the best of our knowledge, ours is one of the largest collections of patients with RMDs submitted to anti-SARS-CoV-2 antibody testing after natural infection, excluding seroprevalence studies. The majority of patients mounted an appropriate IgG response 3-to-11 months post-infection. This is remarkable, considering that most of them were on glucocorticoids and/or DMARDs, including two patients on rituximab. Nonetheless, seroconversion rates were still lower than in controls. The same seems to apply for vaccination response, as reported in a recent SLR by Kroon et al. (32). The proportion of RMDs' patients with a detectable antibody response after an mRNA vaccine against SARS-CoV-2 ranged from 62 to 100% (median 88%, *n* = 8 studies), contrasting to 96–100% (median 100%, *n* = 5 studies) for controls. Curiously, however, we could not find differences in antibody titers between patients and controls after natural infection, whereas all studies analyzed in the SLR (32) demonstrated lower IgG titers or neutralizing titers after vaccination. This suggests that humoral responses in patients with inflammatory RMDs may differ following natural infection or immunization.

These results are encouraging, since they show that having a RMD disease or being on immunomodulators does not seem to greatly influence humoral immune response against SARS-COV-2. Previous reports with limited sample sizes are in agreement with these findings. D'Silva et al. (35) reported that 10/13 (76.9%) RMD patients had detectable anti-SARS-CoV-2 antibodies 7-to-216 days post-infection. Similar results were reported for SLE patients (*n* = 24/29, 83% IgG+), 63% of whom treated with immunomodulators (36). Also, in a larger study recently published by Boekel et al. (37) 120/156 (77%) patients with RMD had positive anti-RBD antibodies at least 6 months after disease onset, and antibodies titers were comparable for all treatment groups, although it was noted a trend toward diminished seropositivity for all bDMARDs (OR: 0.38, 95% CI: 0.18–0.82, *P* = 0.014) and B-cell targeting agents individually (OR: 0.10, 95% CI: 0.010–1.08, *P* = 0.058).

In our study, we found that symptomatic COVID-19 was associated with a higher likelihood of developing a humoral response, and that patients on TNFi tended to have reduced odds of seroconversion. A deleterious effect of TNFi on humoral response to vaccines such as hepatitis B virus has been reported in patients with RMDs (38, 39). Additionally, Kennedy et al. (40) found that patients with inflammatory bowel disease treated with infliximab, exhibited lower seroconversion rates (48 vs. 83%, *P* = 0.00044) and lower magnitude of anti-SARS-CoV-2 reactivity (median 0.8 cut-off index vs. 37.0, *P* < 0.0001), than patients treated with vedolizumab (37, 40). As discussed, TNF is among the major players in the immune response against SARS-CoV-2. Further, a more robust antibody response has been associated with more severe forms of COVID-19 (19). As such, and in line with our results, targeted anti-cytokine therapies, such as TNFi, may, on the one hand, be protective of infection and worse disease outcomes, but, on the other, preclude a proper humoral response (19, 27).

Furthermore, although we did not perform serial antibody testing, we were able to show that sample timing did not influence seroconversion or antibody titers, with titers as high as 1/25,600 up to 331 days after disease onset. Even though it remains unclear what is the optimal antibody titer to protect against reinfection, and what is the role of T-cell-mediated response in adaptive immunity, our results are hopeful regarding the immune response of patients with RMDs. At the very least, they suggest that it is not worse than that observed in the general population (8, 41, 42). Indeed, we demonstrated that anti-SARS-CoV-2 antibodies may persist in patients with RMDs treated with DMARDs for at least up to 11 months. This is close to the 13 months recently reported in 1,309 healthcare workers (43). Likewise, Hamady et al. (44) based on a comparison with other coronaviruses, have estimated a natural antibody-mediated protection for SARS-CoV-2 of up to 1–2 years.

Our study has some limitations, namely concerning the relatively small sample of COVID-19+ patients included. As such, the aforementioned findings must be interpreted with caution. Genetic or environmental factors specific to the Portuguese population might also preclude external generalization. Also, we did not take into consideration potential different social behaviors of COVID-19+ and COVID-19– patients while assessing risk factors for infection. Moreover, during this study time-frame, the use of anti-cytokine therapies for COVID-19 was still not a current practice, so we were not able to evaluate their impact. Finally, ours is not a population-based study. Thus, and even if all centers were aware of a specific call to register COVID-19+ patients in the Reuma.pt, we cannot affirm the total number of patients with RMDs affected by COVID-19 in the whole country during the first 6 months of the pandemic.

In conclusion, we conducted a multicenter, nationwide, comprehensive evaluation of COVID-19 in patients with RMDs, aiming to assess risk factors for infection, predictors of severe/critical disease and antibody response. We found that TNFi and tocilizumab reduced the risk of infection. Treatment with TNFi also tended to reduce rates of both seroconversion and severe disease. These findings warrant further confirmation in independent cohorts. On the other hand, older age, general comorbidities and rituximab are associated with increased risk for infection and worse prognosis, in line with previous reports. Finally, most patients with RMDs seem to be able to develop a proper antibody response after COVID-19, particularly if they had experienced symptomatic disease. Our findings are overall reassuring for patients with RMDs, albeit particular caution must be taken in the more vulnerable patient groups.

## Data Availability Statement

The original contributions presented in the study are included in the article/Supplementary Material, further inquiries can be directed to the corresponding author/s.

## Ethics Statement

The studies involving human participants were reviewed and approved by Comissão de Ética do CHULN e CAML—Centro Hospitalar Universitário Lisboa Norte, Lisbon Academic Medical Center, Lisbon, Portugal. The patients/participants provided their written informed consent to participate in this study.

## Author Contributions

All authors listed have made a substantial, direct, and intellectual contribution to the work and approved it for publication.

## Funding

This study was supported by Merck Sharp and Dohme that provided a research grant for the entire project. Regarding funding sources of the laboratory (MV Lab), we acknowledge Sociedade Francisco Manuel dos Santos, Grupo Jerónimo Martins, the European Union Horizon 2020 research and innovation program, Fundação para a Ciência e a Tecnologia, and the project HighSenseCoV2 financed by Programa Operacional Regional de Lisboa of Portugal 2020 through Fundo Europeu de Desenvolvimento Regional.

## Conflict of Interest

The authors declare that the research was conducted in the absence of any commercial or financial relationships that could be construed as a potential conflict of interest.

## Publisher's Note

All claims expressed in this article are solely those of the authors and do not necessarily represent those of their affiliated organizations, or those of the publisher, the editors and the reviewers. Any product that may be evaluated in this article, or claim that may be made by its manufacturer, is not guaranteed or endorsed by the publisher.
