# Supplementary material for: Risk Factors for Infection, Predictors of Severe Disease, and Antibody Response to COVID-19 in Patients With Inflammatory Rheumatic Diseases in Portugal—A Multicenter, Nationwide Study
Source: Front Med (Lausanne). 2022 Jun 13;9:901817. doi: 10.3389/fmed.2022.901817 (PMC9234392; doi:10.3389/fmed.2022.901817)
Supplement: Supplementary file 1 [file Table_1.DOCX]

**Supplementary table 1 – Comparison of comorbidities and treatment between COVID-19+ and COVID-19 - patients.**

|  | **COVID-19+ (N=162)** | **COVID-19- (N=6201)** | **p-value** |
| --- | --- | --- | --- |
| **Comorbidities, N (%)** |  |  |  |
| Arterial hypertension | 42 (26.0) | 870 (14.0) | 0.392 |
| Obesity | 30 (18.5) | 657 (10.6) | 0.839 |
| Cardiovascular disease | 11 (6.8) | 206 (3.3) | 0.479 |
| Diabetes | 9 (5.6) | 193 (3.1) | 0.718 |
| Malignancy | 7 (4.3) | 114 (1.8) | 0.523 |
| Chronic obstructive pulmonary disease | 5 (3.1) | 47 (0.8) | 0.105 |
| Cerebrovascular disease | 5 (3.1) | 43 (0.7) | 0.080 |
| Chronic kidney disease | 6 (3.7) | 31 (0.5) | **0.008** |
| Hyperuricemia | 2 (1.2) | 27 (0.4) | 0.649 |
| Interstitial lung disease | 2 (1.2) | 77 (1.2) | 0.590 |
| Asthma | 2 (1.2) | 55 (0.9) | 1.000 |
| **Ongoing treatment, N (%)** |  |  |  |
| NSAIDs | 30 (18.5) | 1075 (17.3) | 0.691 |
| Glucocorticoids | 70 (43.2) | 2128 (34.3) | 0.371 |
| Hydroxychloroquine | 25 (15.4) | 760 (12.3) | 0.648 |
| Methotrexate | 64 (39.5) | 2422 (39.1) | 0.173 |
| Sulphasalazine | 15 (9.3) | 560 (9.0) | 0.454 |
| Leflunomide | 11 (6.8) | 395 (6.4) | 0.879 |
| Azathioprine | 6 (3.7) | 128 (2.1) | 0.290 |
| Mycophenolate mofetil | 1 (0.6) | 96 (1.5) | 0.533 |
| TNFi | 24 (14.8) | 2598 (41.9) | **<0.001** |
| Tocilizumab | 5 (2.8) | 373 (6.0) | 0.057 |
| Rituximab | 7 (3.9) | 261 (4.2) | 1.000 |
| Ustekinumab | 1 (0.6) | 31 (0.5) | 1.000 |
| Belimumab | 1 (0.6) | 29 (9.5) | 1.000 |
| JAKi | 2 (1.2) | 86 (1.4) | 1.000 |
