# Supplementary material for: Risk Factors for Infection, Predictors of Severe Disease, and Antibody Response to COVID-19 in Patients With Inflammatory Rheumatic Diseases in Portugal—A Multicenter, Nationwide Study
Source: Front Med (Lausanne). 2022 Jun 13;9:901817. doi: 10.3389/fmed.2022.901817 (PMC9234392; doi:10.3389/fmed.2022.901817)
Supplement: Supplementary file 2 [file Table_2.DOCX]

**Supplementary table 2 – Symptoms and laboratory data of COVID-19+ patients**

| **Epidemiologic link, N (%)** | | |
| --- | --- | --- |
| Direct contact | 111 (68.5) | |
| Health care | 19 (11.7) | |
| Travel to high incidence countries | 3 (1.9) | |
| Unknown | 29 (17.9) | |
| **Symptoms, N (%)** | | |
| Cough | 85 (52.5) |  |
| Fever | 77 (47.5) |  |
| Malaise | 71 (43.8) |  |
| Fatigue | 58 (35.8) |  |
| Myalgia | 53 (32.7) |  |
| Headache | 49 (30.2) |  |
| Anosmia | 49 (30.2) |  |
| Dysgeusia | 48 (29.6) |  |
| Dyspnoea | 31 (19.1) |  |
| Diarrhoea | 30 (18.5) |  |
| Thoracic pain | 28 (17.3) |  |
| Odynophagia | 24 (14.8) |  |
| Arthralgia | 24 (14.8) |  |
| Rhinorrhea | 22 (13.6) |  |
| Abdominal pain | 14 (8.6) |  |
| Vomiting | 12 (7.4) |  |
| **Laboratory abnormalities, n/total N** | | |
| Anaemia | 5/53 |  |
| Leukopenia | 16/53 |  |
| Lymphopenia | 30/53 |  |
| Thrombocytopenia | 5/53 |  |
| Elevated transaminases | 15/52 |  |
| Elevated C-reactive protein | 22/51 |  |
| High D-dimers | 21/31 |  |
| Low fibrinogen | 2/26 |  |
| Ferritin>2000 | 4/22 |  |
