# Supplementary material for: Risk Factors for Infection, Predictors of Severe Disease, and Antibody Response to COVID-19 in Patients With Inflammatory Rheumatic Diseases in Portugal—A Multicenter, Nationwide Study
Source: Front Med (Lausanne). 2022 Jun 13;9:901817. doi: 10.3389/fmed.2022.901817 (PMC9234392; doi:10.3389/fmed.2022.901817)
Supplement: Supplementary file 3 [file Table_3.docx]

**Supplementary table 3 -** **Demographic and clinical data of patients with inflammatory and non-inflammatory RMDs with available serology.**

|  | **Overall**  **(n=65)** | **Inflammatory joint diseases^1^**  **(n=40)** | **CTD/Vasculitis^2^**  **(n=25)** | **p value** |
| --- | --- | --- | --- | --- |
| **Age (years), median (IQR)** | 55 (16) | 53 (16) | 58 (15) | 0.232 |
| **Female, N (%)** | 48 (73.8) | 26 (65.0) | 22 (88.0) | **0.047** |
| **Treatment** | | | | |
| No DMARDs | 12 (18.5) | 8 (20.0) | 4 (16.0) | 0.754 |
| Glucocorticoids | 29 (44.6) | 17 (42.5) | 12 (48.0) | 0.798 |
| csDMARDs | 35 (53.8) | 17 (42.5) | 18 (72.0) | **0.024** |
| TNFi | 9 (13.8) | 9 (22.5) | 0 (0.0) | **0.010** |
| Rituximab | 2 (3.1) | 1 (2.5) | 1 (4.0) | 1.000 |
| Other b/tsDMARDs | 4 (6.2) | 3 (7.5) | 1 (4.0) | 1.000 |
| **COVID-19 severity, N (%)** | | | | 0.523 |
| Asymptomatic^3^ | 8 (12.3) | 5 (12.5) | 3 (12.0) |  |
| Mild | 10 (15.4) | 8 (20.0) | 2 (8.0) |  |
| Moderate | 36 (55.4) | 21 (52.5) | 15 (60.0) |  |
| Severe | 10 (15.4) | 6 (15.0) | 4 (16.0) |  |
| Critical | 1 (1.5) | 0 (0.0) | 1 (4.0) |  |
| **Sample timing**^4^  **(days), median (IQR)** | 237 (149) | 258 (133) | 223 (150) | 0.543 |
| **Seroconversion (N,%)** | 56 (86.2) | 33 (82.5) | 23 (92.0) | 0.463 |
| **IgG titers GM±GSD (min.-max)** | 1/1329±4.063 (1/100-1/25600) | 1/1436±3.96  (1/100-1/25600) | 1/1367±4.262  (1/100-1/25600) | 0.051 |

1 – Includes rheumatoid arthritis, psoriatic arthritis (PsA), spondyloarthritis other than PsA, RS3PE, undifferentiated arthritis and microcrystalline arthritis, adult onset Still disease. 2 – Includes systemic lupus erythematosus, undifferentiated connective tissue disease, mixed connective tissue disease, systemic sclerosis, Sjögren syndrome, giant cell arteritis, Behçet disease; 3 – reference for statistical analysis; 4 – relative to symptom onset or first positive RT-PCR test if asymptomatic. CTD – connective tissue diseases; GM: geometric mean; GSD: geometric SD factor; TNFi: tumour necrosis factor inhibitors.
